# Can signal delay and advertising lead to profit? A study on sporting

**DOI:** 10.3389/fpsyg.2022.1028117

**Published:** 2023-01-17

**Authors:** Yannian Wu, Brian H. Yim, Chaoyun Lu, Luke Mao, James J. Zhang

**Affiliations:** ^1^Institute of Industrial Economics, Jinan University, Guangzhou, Guangdong, China; ^2^School of Foundations, Leadership and Administration, Kent State University, Kent, OH, United States; ^3^Department of Public Finance and Taxation, College of Economics, Jinan University, Guangzhou, China; ^4^Department of Health, Exercise and Sports Sciences, University of New Mexico, Albuquerque, NM, United States; ^5^Department of Kinesiology, University of Georgia, Athens, GA, United States

**Keywords:** streaming media, live sporting event streaming, profit model, advertising, delay

## Abstract

**Introduction:**

Live sporting event streaming (LSES) is becoming popular not only among consumers but also among sponsors. At the same time, influenced by China’s convenient mobile terminals, the paid membership system for live broadcasting has also attracted the attention of marketers and scholars. To promote financial sustainability, we analyzed the internal mechanism of profitability in LSES based on stimulus-organism-response (SOR) theory and two-sided market characteristics. Specifically, we considered advertisement and delay the stimuli (S), arousal and attention as the organism variables (O), and intention to become a paying member as the response (R).

**Methods:**

We used an online survey questionnaire to collect data from 430 Chinese LSES viewers during the 2021 European Cup. We used SPSS Amos v. 26 to conduct structural equation modeling (SEM) and bootstrapping to test the model.

**Results:**

The results show that the direct paths from advertisement and delay to behavioral intention were not significant and that these relationships only became significant via the mediating variables of arousal and attention. Compared to advertising, delay had a stronger indirect effect on behavior. Arousal and attention generated a chain intermediary mechanism in which the presence of attention was necessary.

**Discussion:**

First, LSES platforms should follow Internet development trends and create higher economic value by using precise advertising strategies. Second, LSES platforms should make full use of 5G mobile communication technology to maximize profit. Third, LSES platforms must pay attention to the intermediary mechanism of arousal and attention. Streaming media must provide high-quality events in order to keep target audiences excited.

## Introduction

The Chinese streaming market for sporting events has grown rapidly in recent years. According to a survey conducted by China Internet Network Information Center (CNNIC) (2021), by June 2021, the number of Internet users in China will reach 1.011 billion; among them, the number of consumers of live sports will be 246 million. In October 2014, the State Council of China promulgated “Several Opinions on Accelerating the Development of the Sports Industry and Promoting Sports Consumption,” which clearly requested a relaxation of restrictions on the broadcasting rights for sporting events. One outcome of this report is that CCTV now owns the television (TV) broadcasting rights for Euro 2020. Moreover, using a combination of the free and paid profit models, China Media Group Mobile, IQIYI Sports (merger of Xinying Sports and IQIYI), and Migu Video have the live streaming rights for the event.

Previous findings have drawn attention to the business models of various streaming platforms. Hutchins et al. (2019) defined streaming program providers (e.g., Netflix, YouTube, and Amazon Prime) as over-the-top (OTT) service providers. Although scholars generally describe OTT services as premium services (Kwak et al., 2021), OTT services in China are a mix of free and premium services. By default, sporting events are free for most users and funded by ad revenue, primarily due to weak intention to subscribe to online video services (Kim et al., 2017b). To increase revenue and profitability, some platforms offer subscriptions that significantly improve broadcasting quality. This combination of paid and free viewing to maximize profit is similar to the “freemium” service model in the telecommunications industry. In this model, free users can access content and services, but certain premium features are only available to paid subscribers (Sujata et al., 2015). Nevertheless, the strategy used by Chinese streaming platforms is distinct from traditional telecommunication firms industry and, therefore, are not one-dimensional “freemium” or paid OTT providers.

In the current study, we conceptualized sporting event content providers (e.g., China Media Group Mobile, iQIYI Sports, Migu Video) as Live Sporting Event Streaming (LSES) platforms. In China, most LSES platforms offer both subscription and free options, combining traditional paid services and free services. To maximize profit, platforms try to increase the number of paid viewers because the marginal revenue is significantly higher for new paid subscribers than ongoing free viewers. Some scholars have used analytic frameworks to understand this business strategy. Based on Peitz and Valletti (2008), Calvano and Polo (2020) tested a model for analyzing how a differentiating strategy might satisfy both the subscriber market and the free viewer market. They found that a separate operation for each market would maximize revenue for the platform and that an intentional differentiation strategy for subscribers and free viewers would enhance profitability.

Although this differentiation might call for a brand new design, the traditional model of the two-sided market remains a good option. Scholars have examined multi-sided markets since Rochet and Tirole (2003). However, the behavior of such platforms varies across industries. For online broadcasting, the major LSES platforms in China collect revenue from a two-sided market. LSES platform operation is similar to the two-sided market of football clubs described by Budzinski and Satzer (2011). With viewers who are willing to watch the match by paying a premium price and advertisers who pay to improve their public image and media presence, LSES platforms, as intermediaries, profit by connecting demand from both sides. Armstrong (2006) argued that cross-network externalities exist on both sides of the platform: positive network externality moves from audience demand to advertiser demand, and negative network externality moves in the reverse direction. Therefore, by placing ads in programs and receiving revenue from advertisers, LSES platforms can offer live streaming at a low price or even free of charge.

The business model of LSES platforms differs from traditional sports businesses. Unlike football clubs and other industries, in the LSES industry, quality differentiation primarily depends on signal delay and the amount of advertising. As a result, platforms will increase signal delay and advertising amount to encourage free viewers to become paid subscribers, who enjoy better streaming quality. For free viewers, the platform attempts to increase advertising revenue, offering programs with plenty of ads, often together with a delayed signal, leading to a delayed outcome. Advertising and signal delay, in turn, negatively affect the viewing experience of free viewers, potentially driving them to become paid subscribers. In contrast, for paid subscribers, the platform ensures more fluent and real-time streaming by removing all ads and providing extra bandwidth. Such a differentiation strategy is similar to the one used in the TV broadcasting industry (Calvano and Polo, 2020). Due to the two-sided nature of the market, heterogeneity of programming is necessary to differentiate audiences for the purpose of profit maximization. This strategy has been successful for TV broadcasting, but LSES operation has consistently depended on signal delay and advertising amount to drive free viewers to pay for subscriptions.

Moreover, the mechanism through which services differentiate between free and paid viewers creates psychological and emotional outcomes that require further research. Some scholars have addressed the issue. Turley and Shannon (2000) and Chan and Yeung (2003) found that signal delay and advertising led to disutility among LSES audiences, yet the effect of such disutility on LSES-related purchasing remains unknown.

To address this uncertainty, we examined the attitude of LSES audiences towards delay and advertising using the stimulus-organism-response (SOR) framework (Mehrabian and Russell, 1974). We considered ads and delays as external stimuli (S) that viewers perceive when watching live sport broadcasts, the viewer as the organism (O) influenced by the negative network externalities and psychological effects of ads and delays, and the emotional changes that lead viewers to become paid subscribers as the response (R). Therefore, the purpose of this study was (a) to examine the effect of Advertisement and delay on the intention to become a paying subscriber and (b) to explore the mediating role of arousal and attention in this effect.

The remainder of the paper is organized as follows: literature review, theoretical framework, and hypotheses are provided in Section 2; Section 3 describes the methodology; Section 4 contains the results; Section 5 provides discussion; Section 6 suggests implications; and section 7 provides limitations and suggestions for future research.

## Literature review

### SOR model

As the founder of behavioral psychology, Watson (1913) believed that behavior is an emergency response of the brain to stimuli and that all complex human behaviors can be explained by stimulation and response. This theory posits that behaviors are responses to stimuli but does not account for internal factors. Mehrabian and Russell (1974) proposed an extended consumer stimulus response model (i.e., S-R model) in the book Shopping Behavior Theory. The model explains consumer shopping behavior through four factors: internal and external input (stimulus) and output (reaction). (Belk, 1975) proposed SOR based on previous studies. This classic theoretical model, which explains consumer behavior more widely by accounting for psychological and behavioral changes when individuals encounter stimuli, also applies to behavioral changes in the business environment (Bitner, 1992). Jacoby (2002) pointed out that consumer behavior is an emotional response to external stimuli experienced by an individual, in turn generating consumption behavior. Jang et al. (2020) analyzed previous studies featuring SOR as a theoretical basis for examining how sportscape factors stimulated positive emotions to improve attendance.

With the development of streaming media and changes in consumption, SOR also applies to the purchasing behavior of streaming media consumers. SOR also applies to the purchasing behavior of streaming media consumers. For example, Wei (2013) used the model to explain impulse purchase behavior in an online community environment. Similarly, He et al. (2019) adopted SOR to study the influence of live online shopping on impulsive consumption. As external stimuli, the features of streaming media can also stimulate customers to produce positive or negative emotional responses, in turn influencing purchasing behavior. Scholars have extensively used SOR in sport media research (Breuer et al., 2021; Herold et al., 2021). Breuer et al. (2021) found that when fans watched live football, high game outcome uncertainty increased the magnitude of arousal and emotional response; at the same time, the data show that low-to-moderate arousal and valence-neutral emotional states increased viewer attention to sponsor messages. Herold et al. (2021) examined the network effects of sponsors and viewers on both sides of a professional football club platform on TV viewers’ emotional arousal and attention to sponsor information during live football broadcasts.

### Two-sided market of LSES

A two-sided market arises (Rochet and Tirole, 2006) when a platform serves two different but interrelated customer groups (Evans, 2003). Indirect network externalities connect these two sides; that is, the platform can allow one group to be internalized by the externalities created by another group (Budzinski and Satzer, 2011). LSES platforms in two-sided markets serve two types of users: audiences and advertisers. On the one hand, platforms can provide a series of customized services. While providing some content for free, they also provide subscribers with premium features. From the perspective of subjectivism, streaming platforms meet the viewing needs of most audiences and respect their personal preferences. On the other hand, platforms pay large amounts of money to purchase broadcasting rights for sporting events and sell advertising spots to businesses. This sponsorship allows audiences to watch events for free and identify with sponsoring brands emotionally. These two groups are distinct from each other but related through typical externalities. Testing the relationship between these two groups on streaming media platforms, Herold et al. (2021) found that watching a live football match increased audience enthusiasm. Arousal is an indicator of audience attention to live sporting events and a measure of audience attention to product placement. Therefore, we used SOR in the current study to make predictions about two intermediary variables (i.e., arousal and attention) and develop a theoretical model. We used ads and delays as external stimuli (S), emotional and psychological impact of these stimuli as organism reaction (O), and intention to become a paying member as response (R).

### Advertisement and paying behavior

One feature that LSES platforms often use to differentiate their products is advertising. Youn and Kim (2019) and Kim et al. (2017a, b) found that interstitial advertising (i.e., interactive full-screen ads that cover the interface of a hosting app or a webpage) can induce psychological resistance and behavioral change in customers. In this way, higher amounts of advertisement can negatively affect the purchasing behavior of viewers. Turley and Shannon (2000) tested the relationships among ad recall, purchase intention, and actual purchasing behavior during sporting events and found that ads promoted actual consumption. Kind et al. (2007) found that in a mixed oligopoly market, monopoly platforms can play high-level ads without driving audiences away. However, using two-sided market theory, Kind et al. (2005) found that ads in the monopoly market of traditional media had a negative effect on audiences and then analyzed three profit models of paying, non-paying and mixed paying audiences. Anderson and Coate (2005) investigated the impact of advertising on audience welfare during free or paid live TV watching. The results show that the optimal number of events and ads depended on audience aversion to advertising. In addition, Kind et al. (2005) found that the more intense the competition among media channels, the more stable the advertising revenue. Dietl et al. (2013) found that if the negative effect of advertising was large enough, platform profit increases. Based on previous findings, we predicted that during LSES, ads would elicit an emotional response leading audiences to become paid subscribers. Therefore, we proposed the following hypothesis:

*H1*: Advertisement will positively relate to attitude toward paying member.

### Delay and paying member

When designing webcast payment systems, platforms did not fully understand user preferences related to signal delay (Chan and Yeung, 2003). In fact, streaming services can learn about these preferences using various techniques. For example, by offering different levels of subscription pricing based on maximum tolerable delay, they can collect data about consumer choices. In view of delay preference, Chan and Yeung (2003) proposed a technical processing scheme called “perceived livestreaming delay” to make streaming distribution more efficient for servers. Subscribers who pay more (i.e., advanced users) prefer lower delay and expect lower loss rate. Kooij et al. (2014) found that delay differences between different technologies used to stream events had negative effects, even when the difference was a single second. Therefore, we predicted that during LSES, delays would elicit an emotional response leading audiences to become paid subscribers. This direct relationship between S and R was empirically identified in the previous literature (Luo et al., 2020; Liu et al., 2022; Nguyen-Viet, 2022). Liu et al. (2022) examined the sports live streaming viewers’ perceived value’s (S) effect on their virtual gifting behavior (R). In a green marketing study, Nguyen-Viet (2022) found that the green advertising and eco-labeling (S) have direct positive purchase intention (R). In another green marketing research, Luo et al. (2020) hypothesized green advertising skepticism (S) negatively affects consumer green purchase intention (R). Therefore, we proposed the following hypothesis:

*H2*: Delay will positively relate to attitude toward paying member.

### Arousal

According to Broach et al. (1995) arousal is a state of psychological and physiological activation that a viewer experiences. Emotional response plays a vital role in consumer behavior (Bagozzi et al., 1999; Mao and Zhang, 2013). During exposure to external stimuli such as advertising and signal delay, emotional responses often occur (Jacoby, 2002). Emotions can influence individual cognition, leading to behavioral change. For instance, Shakina et al. (2020) found that audiences were more emotionally than economically driven when purchasing live sports content. Due to the high emotional charge associated with sporting events, the impact of emotion on willingness to pay is more salient (Martin et al., 2008; Biscaia et al., 2012). Among all of the emotional responses elicited by live sports, arousal is pivotal and has attracted much scholarly attention. Arousal, as an emergency response of the human nervous system, is a lower order emotion that is difficulty to control (Zillmann, 1996). Sporting events stimulate a desire to win in audiences, and their level of arousal, in turn, enhances their need for a smooth livestreaming experience (Lantos and Craton, 2012). Therefore, ad interruption might increase willingness to pay.

Cornwell (2019) examined the formation mechanism of network effect through which LSES platforms use ads to generate negative emotions that urge audiences to become paid subscribers. Adelaar et al. (2003) claimed that smooth livestreaming (i.e., no delay) would arouse consumer emotions. Subsequently, Chang and Chen (2008) and Koo and Ju (2010) confirmed that live streaming quality increased level of arousal, in turn affecting purchasing behavior. Therefore, we proposed the following hypotheses:

*H3a*: Advertisement will have a negative effect on arousal.

*H3b*: Delay will have a negative effect on arousal.

### Attention

Another organism-related variable that mediates the link between stimuli and responses is attention. Breuer et al. (2021) found that, in the context of live sports, attention was a mediating factor in the relationship between ad and response and that both color and animation in ads affected viewer attention. During the specific period of a live event, audiences have limited cognitive capacity and, therefore, limited attention to allocate (Lang, 2000). When cognitive capacity is insufficient, viewers might experience cognitive overload. Thus, ads and signal delay during event streaming might lead to dissatisfaction. In addition, Lang (2006) claimed that during LSES, audiences can choose to focus on a wider or narrower area of the screen. The excitement of an event might affect the distribution of attention by expanding or narrowing attentional space. Previous findings show that advertising and delay aroused negative emotions, potentially shifting audience attention (Heath and Nairn, 2005). However, Breuer and Rumpf (2015) and Lee and Faber (2007) claimed that ads are typically peripheral rather than central. Because the information of greatest interest to the audience is the progress and outcome of the competition, signal delay is more likely to arouse attention than advertising. In addition, Kooij et al. (2014) found that an Internet connection with high latency affected audience perception of live events. High latency led to long pauses, arousing negative emotions due to distraction. Therefore, minimizing delay time is essential to satisfactory fan interaction. We predicted that viewers would experience negative emotions and distraction in the presence of advertising and delay. Therefore, we proposed the following hypotheses:

*H4a*: Advertisement will have a negative effect on audience attention.

*H4b*: Delay will have a negative effect on audience attention.

*H4c*: Arousal have a negative effect on audience attention.

### Subscription purchase

Previous findings show that emotional reaction influences impulse purchase behavior during LSES (Lv et al., 2022). Using SOR to test the interference of online store atmosphere on shopping intention, Koo and Ju (2010) found that arousal and attention influenced consumer behavior. Similarly, Chang and Chen (2008) found that consumer emotions mediated the relationship between website quality and purchase intention. Regarding live shopping, Liu et al. (2020) found that arousal significantly affected impulse purchasing. Based on these previous findings, we proposed the following hypotheses:

*H5a*: Arousal will have a negative effect on free viewers to pay member.

*H5b*: Attention will have a negative effect on free viewers to pay member.

### Mediating effects of arousal and attention

Pozharliev et al. (2022) argued that customers exposed to augmented reality advertising are driven by physiological arousal and lead to higher behavior intentions to pay. Goncalves et al. (2022) analyzed consumer responses to different advertisements and examined consumers’ emotional arousal and attention. The results showed that video ads are more effective at capturing consumers’ attention and eliciting more emotion than image ads. Güzel et al. (2020) investigated the influence of emotional arousal on motivation to drive travel behavior and found that emotional arousal mediates this relationship. Guerreiro et al. (2015) demonstrated that physiological responses such as emotional arousal and attention are indeed important predictors of consumer behavior. In addition, the Internet with high delay affected the audience’s emotional arousal, and the pause caused by delay caused negative emotions that negatively affected the audience’s attention (Kooij et al., 2014). Based on the empirical results of the previous literatures, we propose the following hypotheses:

*H6a*: Arousal and attention will be the chain mediating variable between advertisements influencing audience to become paying members.

*H*6b: Arousal and attention will be the chain mediating variable between delay influencing audience to become paying members.

*H*6c: Attention will be the mediating variable between advertisements influencing audience to become paying members.

### Theoretical framework

**Figure 1:**
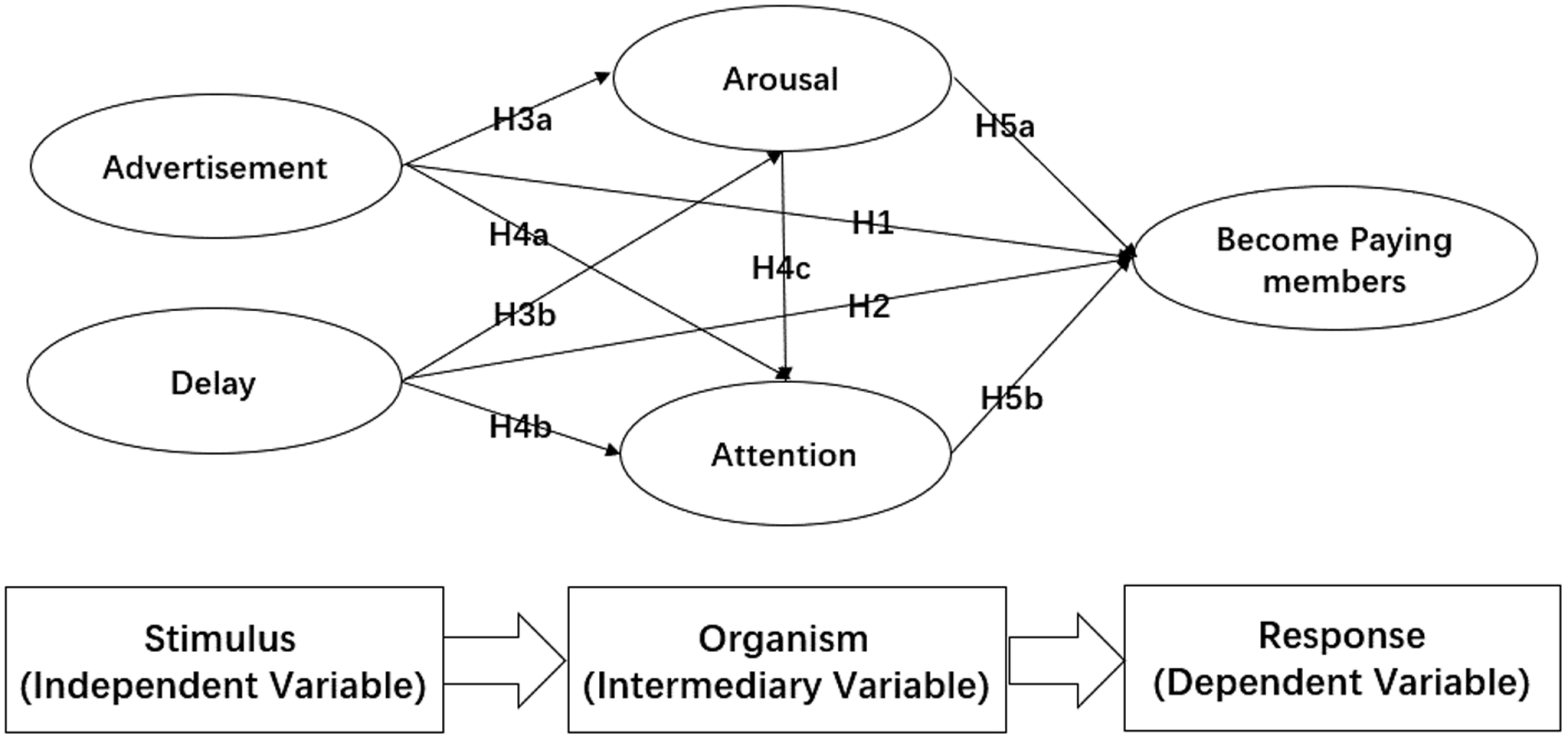
Proposed hypothetical model.

In view of previous findings and based on SOR, we constructed a theoretical model for factors that influence purchase behavior in the context of LSES subscription. Advertising and delay were stimulus factors (i.e., independent variables), audience arousal and attention were intermediary variables, and becoming a paying subscriber was the dependent variable (see Figure 1).

## Materials and methods

### Subject

A pilot study was first conducted to preliminarily examine the measurement properties of the survey questionnaire, which had 60 LSES viewers in China who responded to the survey administration during the first round of the 2021 European Cup group *via* WeChat, the primary social media platform in China. For the actual investigation to examine the research questions, research participants (*N* = 430) were also LSES viewers in China. Survey administration was conducted in large, medium, and small cities across China, including Beijing, Shanghai, Guangdong, Shanxi, Guangxi, Hunan, Hainan, and Xinjiang, through the online survey platform “Questionnaire Star” (see Table 1). In order to screen the LSES viewers of the 2021 European Cup, we asked a filtering question at the beginning of the survey “Have you watched 2021 European Cup streaming media event?”

**Table 1:** Demographic characteristics of the respondents.

Characteristics		(*n* = 430)	%
Gender	Male	310	72.09%
	Female	120	27.91%
Age	Under 18	18	4.19%
	18–22	259	60.23%
	23–35	83	19.3%
	36–45	46	10.7%
	Above 45	24	5.58%
Education background	High school and below	17	3.95%
	College	18	4.19%
	Undergraduate	305	70.93%
	Master	64	14.88%
	Doctor	26	6.05%
Monthly consumption **(RMB)**	Less than 1,000	82	19.07%
	1,001–3,000	223	51.86%
	3,001–5,000	47	10.93%
	More than 5,000	78	18.14%
Years of using streaming media	1 year or less	133	30.93%
	2 years	43	10%
	3 years	52	12.09%
	4 years	22	5.12%
	5 and above	180	41.86%

### Measurements

The scales used in this study were adapted from existing studies to fit the Chinese LSES context. To ensure the consistency of the measurement tools, we used reverse translation to proofread the content of the survey questionnaire in English and Chinese. All of the items were measured on a 5-point Likert-type scale (see Table 2).

**Table 2:** CFA results for measurement model (*n* = 430).

Dimension	λ	α	CR	AVE
Advertisement		0.952	0.952	0.870
(1) Advertisements are usually annoying when I’m watching live sports streams	0.925			
(2) Advertisements are usually irritating when I’m watching live sports streams	0.950			
(3) Advertisements usually make me angry when I’m watching live sports streams	0.923			
Delay		0.962	0.963	0.897
(1) I lose interest because of the time delay when I’m watching live sports streams	0.957			
(2) I lose interest because of the unsmooth picture when I’m watching live sports streams	0.967			
(3) I lose interest because of the unclear picture when I’m watching live sports streams	0.916			
Arousal		0.939	0.941	0.840
(1) Live sports streams with advertisements and delays make me fidget	0.900			
(2) Live sports streams with advertisements and delays make me impatient	0.943			
(3) Live sports streams with advertisements and delays make me anxious	0.907			
Attention		0.946	0.947	0.856
(1) Live sports streams with advertisements and delays make it hard for me to focus on sports stars	0.924			
(2) Live sports streams with advertisements and delays make it hard for me to focus on the score	0.933			
(3) Live sports streams with advertisements and delays make it hard for me to focus on the results	0.918			
Paying behavior		0.939	0.940	0.840
(1) I’m willing to pay for a subscription to watch live sports streams without advertisements or delays	0.860			
(2) I’m glad to pay for a subscription to watch live sports streams without advertisements or delays	0.962			
(3) I’d rather pay for a subscription to watch live sports streams without advertisements or delays	0.925			

#### Advertisement

In their scale index of advertising stimulation factors in a live streaming media environment, Sigurdsson et al. (2018) defined ad responses as “angry, impatient, or even moderately angry”; we adopted this conceptualization. In addition, we used the scale developed by Xu (2006) to measure the impact of streaming media ads on audience emotion, adopting the proposal that advertising stimulation would trigger negative attitude toward the media and reduce advertising value. Three items were used to measure the impact of Advertisement.

#### Delay

We adapted the live streaming delay scale of Centieiro et al. (2015), who found that when people watch live sports, asynchronous multimedia streams ruined the viewing experience. In fact, prompting users to guess whether a goal would be scored in the next few seconds of a football match revealed that some users were frustrated or stressed because they were unable to perform this operation due to delay. We use three indicators from the scale used by Centieiro et al. (2015).

#### Arousal and attention

We chose a well-established scale to measure the mediating variable “emotional performance,” which we divided into two dimensions: arousal and attention. For arousal, we adopted the Mood Scale (PAD) developed by Russell and Mehrabian (1974) and the scale developed by Mukhopadhyay and Johar (2007). We used the scale classification standard (Yadav et al., 2007), divided attention into external attention (EA) and internal attention (IA), and adapted the questionnaire to fit a Chinese audience. Six items were used to measure arousal (three items) and attention (three items).

#### Paying member

We adapted intention to become a paying subscriber from Jarvenpaa et al. (2000) and Davis et al. (1989). We made appropriate adjustments to fit the consumption characteristics of Chinese streaming media audiences. We measured Paying member using three items.

### Pilot study

Of the 60 respondents who participated in the pilot study, there were 45 males (75%) and 15 females (25%). A majority of them were 18–22 years old (58.333%), followed by 23–35 years old (10%) and 36–45 years old (13.33%). Almost all of them had a college degree (60%) or an advanced degree (33.33%). While over one half of the respondents had been using streaming media for more than 5 years (51.67%), 8.33% of viewers used the service for merely 1 year or less.

Cronbach’s alpha (α) coefficient test was calculated to examine the reliability of the questionnaire. The overall reliability of the questionnaire results was 0.877, indicating that data reliability was very high. The reliability values for each of the constructs contained in the questionnaire were all greater than 0.8, indicating that the reliability of the five dimensions of the independent, mediating, and dependent variables were internally consistent and overall acceptable. It was deemed appropriate to proceed with the study.

### Data analyses

For the actual investigation, procedures in SPSS Amos v. 26 were used to analyze the data. Following the recommendations of Anderson and Gerbing, we conducted data analyses *via* a two-stage approach. In the first stage, we used confirmatory factor analysis (CFA) to test the reliability and validity of the scale. In the second stage, we used structural equation model (SEM) to test the proposed hypotheses. We then used bootstrapping to test the mediating effects.

## Results

### Characteristics of participants

As shown in Table 1, respondents for the actual investigation included 310 males (72.09%) and 120 females (27.91%). Most of them were 18–22 years old (60.23%), followed by 23–35 years old (19.3%) and 36–45 years old (10.7%). A large majority of these LSES viewers had a college degree (70.93%) or an advanced degree (20.93%). A large portion of the respondents had been using streaming media for either more than 5 years (41.86%) or 1 year or less (30.93%).

### Measurement model

The measurement model used maximum likelihood (ML) estimation to test the correlations among the 15 items across 5 latent variables: 3 items for advertisement, 3 items for delay, 3 items for arousal, 3 items for attention, and 3 items for purchasing behavior (see Table 2).

CFA permits assessment of model fit through the following indices: chi-square/*df* < 5.0 (Kline, 2011), root mean square error of approximation (RMSEA) < 0.08 (Kline, 2011), goodness of fit index (GFI) > 0.90, Tucker-Lewis index (TLI) > 0.90 (Hooper et al., 2008), and comparative fit index (CFI) > 0.90 (Hair et al., 2011). In this study, model fit was acceptable (*χ*^2^ = 173.812, *df* = 80, *p* < 0.05, *χ*^2^*/df* = 2.173, RMSEA = 0.052, TLI = 0.984, GFI = 0.950, CFI = 0.988).

We used Cronbach’s alpha (α > 0.70) and composite reliability (CR > 0.70) to test the reliability of the factors. We tested construct validity using convergent validity and discriminant validity. The factor loadings (*λ*) of all measurement variables were higher than the critical standard of 0.50 and significant, and average variance extracted (AVE) was higher than 0.50, indicating that the scale had convergent validity (Hair et al., 2014). Discriminant validity derives from the fact that the square root of AVE is greater than the correlation between structures (Fornell and Larcker, 1981). Table 2 shows that all Cronbach’s alpha values exceeded the minimum reliability standard of 0.7 recommended by Nunnally and Bernstein (1994). The CR value ranged from 0.940 to 0.963, which exceeded the threshold of 0.70 (Hair et al., 2014). The factor loading (*λ*) of each measurement variable was higher than the critical standard of 0.50, and the range was 0.860 ~ 0.962. AVE values ranged from 0.840 to 0.897 exceeding the threshold of 0.50 (Lin et al., 2020). The model had sufficient reliability and convergence validity.

To test discriminant validity, we followed Fornell and Larcker (1981) by comparing the positive square root of AVE with the absolute values of correlation coefficients among the constructs. Table 3 shows that the correlation coefficient (0.263 ~ 0.836) of each variable was less than the square root of the AVE of each variable (0.872 ~ 0.947), indicating discriminative validity. In general, the measurement model had sufficient discriminant validity.

**Table 3:** Discriminant validity of the model.

	Advertisement	delay	Arousal	Attention	Become paying members
Advertisement	0.872				
Delay	0.809***	0.947			
Arousal	0.501***	0.539***	0.917		
Attention	0.487 ***	0.567***	0.836***	0.925	
Paying members	0.263***	0.265***	0.345***	0.383***	0.917

The diagonal elements are the square root of AVE.

### Structural model

After validating the measurement model, we tested SEM to verify the nomological relationships (Alexandris et al., 2011) among the five variables: (a) direct relationships between paying members and advertisement, delay, arousal, and attention, respectively, (b) direct relationships between arousal and both advertisement and delay, respectively, (c) direct relationships between attention and both advertisement and delay, respectively, (d) and mediating effect of arousal and attention in the respective relationships between the two independent variables and paying members.

The indices used to evaluate the measurement model indicated structural model fit: *χ*^2^*/df*, RMSEA, GFI, TLI, and CFI. The model fit indices revealed that the measurement model and the structural model were acceptable and that degree of fit was very good: *χ*^2^/df = 2.17, RMSEA = 0.052, TLI = 0.980, and CFI = 0.990. The five latent variables in the measurement model had strong reliability and validity.

We analyzed the structural model to test the H1–H4. The independent variables did not significantly influence to pay member directly, leaving H1 and H2 unsupported. Arousal was significantly influenced by advertisement (*β* = 0.188, *t* = 2.379) and delay (*β* = 0.386, *t* = 4.884), supporting H3a and H3b. Attention related to delay (*β* = 0.211, *t* = 3.696) and arousal (*β* = 0.752, *t* = 17.613), supporting H4b and H4c, but advertisement, leaving H4a unsupported. Attention significantly influenced paying member (*β* = 0.296, *t* = 2.951), supporting H5b, but arousal did not significantly influence this Behavior, leaving H5a unsupported (see Table 4).

**Table 4:** SEM results for structural model.

Path coefficients between factors	*β*	*T*	Hypothesis
H1: Advertisement →paying members	0.107	1.232	Not Supported
H2: Delay →paying members	−0.018	−0.202	Not Supported
H3a: Advertisement → Arousal	0.188	2.379*	Supported
H3b: Delay → Arousal	0.386	4.884***	Supported
H4a: Advertisement → Attention	−0.060	−1.087	Not Supported
H4b: Delay → Attention	0.211	3.696***	Supported
H4c: Arousal → Attention	0.752	17.613***	Supported
H5a: Arousal → paying members	0.054	0.553	Not Supported
H5b: Attention →paying members	0.296	2.951**	Supported

*p < 0.05; **p < 0.01; ***p < 0.001.

We used bootstrapping to test the mediating effects of arousal and attention based on 5,000 samples. Bootstrapping provides a confidence interval for indirect effects, indicating mediating effects when the interval between the lower and upper bounds of the 95% confidence interval does not contain zero (Byrne, 2016). The mediating effects of arousal and attention were significant because the (95%) interval did not contain 0 in any of the paths. In LSES, the path “Advertising →Arousal → Attention→ paying member” was significant; the confidence interval ranged from 0.003 to 0.100, and the indirect effect value was 0.045, *SE* = 0.024, *p* = 0.035 < 0.05. The chain mediating effect of “Advertisement →Arousal → Attention→ paying member” was significant; the proportion of mediating effect was 21.7%, the confidence interval ranged from 0.054 to 0.168, and the indirect effect value was 0.104, *SE* = 0.029, *p* = 0.000 < 0.01. The indirect effect of the path “Delay → Arousal → Attention → paying member” was significant; the proportion of mediation effect was 50.1%, and the confidence interval ranged from 0.020 to 0.100. The indirect effect of the path “Delay → Attention →paying member” was significant; the proportion of mediation effect was 28.2%, and the total effect of the mediation was 0.207. The effect percentage of delay on paying member was 78.3%, indicating that delay had a major influence on the target behavior (see Table 5).

**Table 5:** Bootstrapping test for mediating effects.

Indirect effect	Effect size	SE	Percentile 95% CI			Effect ratio (%)
			Lower	Upper	*p*	
H6a: Advertisement→ Arousal → Attention → paying members	0.045	0.024	0.003	0.100	0.035	21.7%
H6b: Delay → Arousal → Attention →paying members	0.104	0.029	0.054	0.168	< 0.001	50.1%
H6c: Delay → Attention →paying members	0.059	0.020	0.024	0.100	0.001	28.2%

## Discussion

The purpose of this study was to explore purchasing behavior among LSES viewers by investigating the interrelationships among advertisement, delay, arousal, attention, and intention to become a paying subscriber. The findings, based on the SOR theory of consumer psychology and the context the two-sided market of LSES platforms, enhance the theoretical understanding of LSES profit models.

The findings show that advertisement and delay did not directly influence paying member, a result that conflicts with previous findings (Turley and Shannon, 2000; Chan and Yeung, 2003). Two reasons explain this inconsistency. On the one hand, in previous studies, scholars did not distinguish between direct and indirect relationships among advertisement, delay, and purchasing behavior. On the other hand, audiences usually engage in cognitive evaluation on ads and delays when streaming live sporting events but do not directly engage in purchasing behavior. From the perspective of consumer behavioral psychology, purchasing behavior typically follows a three-stage process: cognition → emotion → behavior. Therefore, cognitive changes due to ads and delays are not likely to induce purchasing directly.

We introduced SOR theory and conducted chain mediation testing through AMOS, SEM, and bootstrapping. We found that the chain mediation effect of the indirect path of “Advertisement → Arousal → Attention → paying members” was significant, accounting for 21.7% of the total effect. We found an indirect relationship between advertisement and paying members; that is, advertisement during live sports triggered negative evaluation, aroused negative emotions, and shifted attention away from primary content.

The results indicate that the indirect effect of the path of “Delay → Arousal → Attention → paying members” was significant. The chain of intermediary effect accounted for 50.1% of the total effect, suggesting that streaming delays also aroused negative emotions, distracting them from the game and, eventually, encouraging them to pay for live sports streaming. In general, the indirect effect of signal delay on intention to become a paying subscriber reached 78.3%, indicating that delay had a great indirect impact.

In most previous studies, scholars examined purchasing behavior based on the assumption of an external mechanism and a rational audience, not accounting for consumer psychological factors. In the current study, we found that two emotional reactions (i.e., arousal and attention) were important intermediary factors in become paying members. In the two-sided market of LSES, advertisement and delay, as external stimuli, likely do not directly lead audiences to pay for subscriptions but can induce that behavior through the intermediary effect of arousal and attention. This finding shows that the mechanism for purchasing behavior is likely the following: “external stimulus-cognitive change-emotional change-behavioral change.” That is, the two external factors (i.e., advertisement and delay) stimulated cognitive change, inducing emotional response and, in turn, encouraging the audience to pay for a subscription. In addition, Zillmann (1996) pointed out that arousal was an uncontrollable low-level emotion and an automatic emergency response of the central nervous system during exposure to external stimuli. We found that arousal did not significantly affect behavioral intention, further demonstrating that arousal requires the mediating effect of attention. Therefore, in order to encourage more people to become paid subscribers, LSES platforms should consider the mechanism of purchasing behavior when considering ad production and delivery and providing high-definition, non-delayed content.

## Conclusion and implications

### Conclusion

Based on SOR, the results suggest the following conclusions:

Advertisement and delay did not directly induce the audience to pay member but required intermediary variables.

1. In the mediated pathways, the impact of delay was stronger than advertisement. Therefore, delay might better stimulate induce the audience to pay for a subscription.
2. In the mediated pathways, arousal was not strong enough without attention to induce the audience to pay member.

### Implications

Due to COVID-19, major sporting events and professional leagues are not fully open for live attendance, highlighting the importance of livestreaming for sports organizations. Moreover, many events occur simultaneously, and livestreaming allows fans to choose which to watch. Whether to pay for premium features is an important choice for LSES audiences. Therefore, our findings have several important implications for LSES platforms, especially during a situation that prevents live attendance.

First, LSES platforms should follow Internet development trends and create higher economic value by using precise advertising strategies. Along the way, they should pay more attention to the social value of free live sports. Based on SOR, our findings verify that ads can induce the audience to become paid subscribers through the indirect effect of attention and that the network cross-externality effect between advertising and audience plays a role in LSES. Therefore, LSES platforms can gain more control over the consumption habits and viewing preferences of target audiences through Internet technology and big data (e.g., analysis of viewing time, gender, age, consumption ability, supported teams, and favorite sports stars) to improve advertising accuracy. In addition, they can “tailor” ads to the events preferred by consumers, minimizing audience disgust and maximizing economic benefit. This recommendation is particularly applicable to LSES in China. According to the regulations of the State Administration of Press, Publication, Radio, Film and Television, China Central Television (CCTV), a non-profit institution, buys the copyrights to broadcast the Olympics and other major international sporting events so that more viewers can watch for free. Under these conditions, for the Olympic Games, Asian Games, and other events, LSES platforms should focus on the “paid advertising + free audience” profit model to highlight their social value.

Second, LSES platforms should make full use of 5G mobile communication technology to maximize profit. 5G ultra broadband technology enables LSES platforms to accommodate larger audiences. These include an increase in paid and free audiences. On the one hand, when the number of free audiences increases, LSES platforms can attract more advertisers, thereby increasing revenue. on the other hand, the higher the frequency of clicking to watch, the greater the economies of scale effect, and the lower the actual cost of live streaming. At the same time, under the positive external effect, audiences will pay more attention to live games. For example, streaming media can enhance the consumer experience by enabling full live delivery of high-rate, low-latency, 4 K ultrahigh-definition streaming through 5G technology, as well as experimenting with 8 K ultrahigh-definition delivery, potentially inducing willingness to pay member. Overall, LSES platforms need to innovate and integrate the external incentives of streaming media through 5G technology, make audiences focus on watching the primary content, and effectively strengthen willingness to pay in order to increase profit margins.

Third, LSES platforms must pay attention to the intermediary mechanism of arousal and attention. Streaming media must provide high-quality events in order to keep target audiences excited. The underlying mechanism lies in the fact that a dull game lowers arousal while a good game increases arousal. The greater the stimulation of the game, the stronger the audience’s visual attention, and a bland live game is likely to cause viewers to pay more visual attention to ads. Therefore, the streaming media need to offer a captivating picture during broadcasting. Finally, in the decision to influence audience payment, the streaming media should always try to attract audience attention. Using rich event resources is likely to attract the attention of more viewers, increasing potential customers for advertisers and potential subscribers.

## Limitations and future research

The current study has several noteworthy limitations: First, the questionnaires were distributed mostly to new media audiences in big cities. Scholars should expand the scope and objective of the survey to include urban and rural customers. Second, the factors influencing new media live sports broadcasting are various. Scholars should consider how uncertainty about and interaction with the results of sport competitions might influence purchasing behavior. Third, LSES platforming is a multilateral market and involves many cognitive and emotional reactions. Scholars should investigate other mediating and moderating variables. Fourth, our research object was new media platforms. A comparative analysis of old and new media platforms is likely to reveal some differences willingness to pay for premium services. Fifth, arousal and valence are two dimensions of emotion, and future research will address an important feature of emotion that has not been studied in depth in this study, namely the dimension of valence. Lastly, in the current study we did not measure the actual paying for membership behavior but the intention to become a paying member. Future study may conduct a longitudinal study to measure the actual paying membership behavior.

## Data availability statement

The raw data supporting the conclusions of this article will be made available by the authors, without undue reservation.

## Ethics statement

The studies involving human participants were reviewed and approved by the study was conducted according to the guidelines of the Jinan University, and the Ethical review and approval were waived for this study as it was considered a Level 1 study (covers research with participants that is ‘non-problematic’). The patients/participants provided their written informed consent to participate in this study.

## Author contributions

YW contributed conceptualization, literature review, methodology, and analysis, and original draft preparation. BY contributed the introduction, result interpretation, methodology revision, and manuscript editing. CL contributed the discussion and manuscript editing and oversaw the research design and execution and contributed to the manuscript composition. LM contributed the document preparation and methodological guidance and manuscript structuring and revision and concept clarification and determination of nomenclature. JZ contributed the literature review and research quality control. All authors read and approved the final manuscript.

## Funding

We received funding from Guangdong Province Philosophy and Social Science Planning Project. Approval number: GD19CTY08.

## Conflict of interest

The authors declare that the research was conducted in the absence of any commercial or financial relationships that could be construed as a potential conflict of interest.

## Publisher’s note

All claims expressed in this article are solely those of the authors and do not necessarily represent those of their affiliated organizations, or those of the publisher, the editors and the reviewers. Any product that may be evaluated in this article, or claim that may be made by its manufacturer, is not guaranteed or endorsed by the publisher.
